# Cardiac Screening in Young Athletes: The Role of Diagnostics in Preventing Sudden Cardiac Death and Exploring Clearance Protocols

**DOI:** 10.3390/jcm15051895

**Published:** 2026-03-02

**Authors:** Ala W. Abdallah, Darren Nguyen, Osama Odeh, Noyan Ramazani, Jaineet Chhabra, Nazanin Houshmand, Tahir Tak

**Affiliations:** 1 Department of Internal Medicine, University of Nevada Las Vegas, Las Vegas, NV 89102, USA; ala-abdallah@uiowa.edu (A.W.A.); noyan.ramazani@unlv.edu (N.R.);; 2 Carver College of Medicine, Iowa City, IA 52242, USA; 3 Houston Methodist, Houston, TX 77030, USA

**Keywords:** preparticipation, cardiac, clearance, sports, sudden cardiac death, electrocardiogram, imaging, guidelines

## Abstract

**Background**: Sudden cardiac death (SCD) and sudden cardiac arrest (SCA) remain leading causes of mortality in young athletes, highlighting the importance of cardiac screening prior to play. The guidelines on screening protocol are continually evolving but are often inconsistent across organizations. We explore the role of different screening modalities including electrocardiography (ECG) and various cardiac imaging testing; their effectiveness, cost considerations and clinical utility regarding sports cardiac screening, comparing European and American cardiac screening protocols. Additionally, we also discuss the divergence between the European and American guidelines and appraise the literature surrounding this topic. **Methods**: A comprehensive literature review was conducted using studies published between 2010–2025 on cardiac screening for young athletes. Parameters included an English filter with review of observational studies, systematic reviews, meta-analyses, and randomized controlled trials (RCTs). Manual searches of PubMed, Embase, and Google Scholar libraries were also performed to enhance the reach of our investigation. Articles were chosen based on relevance to the topic. **Results**: When compared to PPCS physical exam, ECG advantages include increased detection of cardiac conditions leading to SCD, improved sensitivity and specificity compared to history alone, Disadvantages are the need for trained specialists to complete testing properly, unreliability for detecting congenital coronary anomalies, cost-effectiveness concerns, and false positive risk. However, these disadvantages can be improved with improved ECG interpretation training for non-cardiologists and policies easing access to advanced cardiac care. **Conclusions**: ECG remains the cornerstone of cardiovascular screening due to its affordability and sensitivity in detecting electrical abnormalities, its limitations necessitate a multimodal approach. Integrating targeted ECG screening with echocardiography and advanced imaging in select cases may enhance diagnostic accuracy while balancing cost-effectiveness and accessibility. While the benefits of routine ECG are strongly supported by the literature, nationwide implementation of it remains challenging due to economic, geographical and logistical restraints. Therefore, more research needs to be conducted on the mortality benefits and cost-effectiveness of routine ECGs implementation in PPCS screening for young athletes.

## 1. Introduction

Cardiovascular disease screening is universally important, not only for decreasing morbidity and mortality in the elderly, but to also reduce the number of deaths within the youth population, especially in athletes and/or those involved in rigorous exercise programs. Athletes are screened for cardiovascular diseases to prevent sequelae such as those related to hypertrophic cardiomyopathy (HCM), arrhythmia, valvular disease, and septal defects [1,2]. PPCS cardiovascular clearance is a routine screening examination that millions of children and adolescents undergo each year to participate in school-sponsored sports. The aim of this screening is to identify high risk individuals for further testing, with the goal of preventing sudden cardiac death (SCD) from occurring to an athlete [3,4]. Unfortunately, if there is an underlying heart disease then exercise and competitive play could be the trigger of SCD [5]. Although this phenomenon is very rare, it has significant consequences for affected families, sports teams, and communities. In essence, the goal of PPCS screening is to identify and risk stratify those with structural or electrical heart disease. However, there remains debate over the best methodology of handling these cases [6]. The purpose of this review is to evaluate the current PPCS screening guidelines, highlight the discrepancies between American and European recommendations, discuss the effectiveness and limitations of various screening methodologies, and explore the economic implications of these screenings. Moreover, this review aims to provide a comprehensive understanding of the etiologies and prevalence of SCD in the US and Europe and the current state of affairs in the prevention of SCD.

## 2. Methods

This article is a narrative literature review. A comprehensive review of the literature was conducted to provide an overview of cardiac screening practices for young athletes. Studies published between 2010 and 2025 were identified through searches of PubMed, Embase, and Google Scholar. Searches were limited to English-language publications and included observational studies, systematic reviews, meta-analyses, and randomized controlled trials (RCTs).

Search terms included combinations of: (“cardiac clearance” OR “cardiovascular screening”) AND (“competitive sports” OR “athletes”) AND (“sudden cardiac death” OR “sudden cardiac arrest”) AND (“guidelines” OR “prevention” OR “etiology”). Study selection was guided by relevance to sudden cardiac death in athletes, the role and importance of preparticipation cardiac screening (PPCS), and the utility, cost-effectiveness, and outcomes associated with electrocardiography (ECG) and cardiac imaging. Additionally, studies that comment on the medico-legal perspective of this topic were also chosen. Emphasis was placed on studies addressing current cardiac screening guidelines and their evolution over time, with comparison of American and European recommendations.

### 2.1. Definition and Epidemiology of Sudden Cardiac Death

Sudden cardiac death (SCD) is defined as the unexpected death of cardiac etiology in a seemingly healthy person, typically occurring less than 1 h after the onset of symptoms [7]. Most cases of SCD and sudden cardiac arrest (SCA) mostly rely on media reports, registries, or reviews of diagnoses and autopsy reports and as a consequence cases can be missed and incidence will appear falsely low [8]. Studies have consistently shown that men have a higher rate of SCD than women with a ratio of 3:1 and the median incidence of SCD was 2.7 per 100,000 person-years (1 in 37,000) [8]. In the US, SCD became a national topic following the high-profile deaths of basketball players Hank Gathers and Reggie Lewis [9,10]. Their sudden deaths during athletic activity had significant ramifications and inspired the first conference to lay down the foundation of PPCS [9,10].

According to the National Collegiate Athletic Association (NCAA), during 2003–2013, the most common medical cause of death among college age athletes (ages 17–23 years) is SCD [11]. The annual incidence was found to be 1 per 53,703 athlete years at the time [11]. Among the studied population, males, blacks, and Division I basketball and football players were demographics associated with the highest rates of death [11]. Additionally, autopsy-negative death was a common finding, occurring in 25% of cases [11]. A more recent NCAA database analysis supports these findings [12]. From the period 2002–2022, a total of 143 SCD cases have been identified [12]. Additionally, annual SCD incidence steadily decreased over the period of the study, but the overall SCD incidence is 1 per 63,682 athlete years [12]. Similarly, Black athletes, as well as Division I basketball players, were the groups most affected [12]. Autopsy-negative SCD was the most common post-mortem finding [12]. Idiopathic left ventricular hypertrophy was the most common structural defect identified [12]. Malhotra et al. investigated the incidence and causes of SCD in the amongst young UK soccer players (mean age 16 years) with H&P, ECG and echocardiography from 1996 to 2016 [13]. It was found that 0.38% of all those screened had a cardiac disorder associated with SCD. HCM and ARVC were the two most common diseases diagnosed [13]. In older athletes (ages > 35 years), coronary artery disease and hypertrophic cardiomyopathy are the most common etiologies of SCD [14]. However, because there are methodical variations in estimating SCD incidence—such as use of different databases, geographic location, age populations, target demographics (race especially) and definition—epidemiological studies often produce varying incidence calculations. As a result, the results of these epidemiological studies are not generalizable for any population outside its studied demographics. For example, conclusions gathered from NCAA databases cannot be applied to European athletic populations due to the differences in demographics and environment. These differences are one key reason while there is divergence in guideline recommendations.

Over time, the overall incidence of SCD has decreased with new guidelines being in place to better screen for and stratify high-risk athletes [12]. Advances such as the ubiquitous presence of automatic external defibrillators (AED) and personnel trained in cardiopulmonary resuscitation (CPR) helped save lives and mitigate the damage caused by SCD [15,16]. The causes of SCD in young athletes remains elusive in the United States as there is no coordinated system for postmortem evaluation but events rely on a patchwork of medical examiners and coroners that can create a variable quality and specificity of autopsies [8]. In other parts of the world, there are dedicated and specialized referral centers where extensive testing can be done but limitations exist in those places and ascertainment bias has high culpability in those places [8]. Regardless of the analytical significance and the descriptive statistics related to the epidemiological impact of SCA and SCD on young athletes, the causative factors of SCA and SCD in the adolescent and young adult population still remains largely debatable. 

### 2.2. Etiologies of Sudden Cardiac Death

The etiology of SCD is diverse, consisting of structural heart disease and inherited channelopathies. Previously, studies show that HCM is the most common cause of SCD amongst young athletes in the US [17,18]. On the other side, arrhythmogenic cardiomyopathy (ACM) in the United Kingdom and Italy [19,20]. However, recent studies now show that SCD in structurally normal hearts is now the most common autopsy finding as previously mentioned [11,12,20,21].

**Figure 1:**
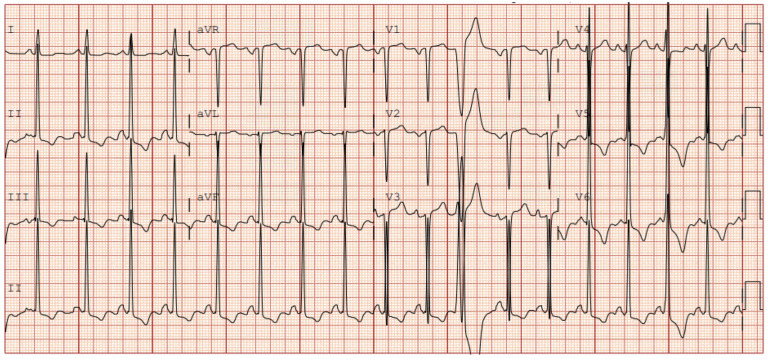
Electrocardiogram tracing of a patient with hypertrophic cardiomyopathy. with significant LVH in leads V1–V6. There are very prominent R-waves in the lateral leads and inferior leads. Additionally, notice the deep S-waves of V1–V3.

HCM is the most common cause of SCD out of all the structural heart diseases. It is an inherited disease of myocardium sarcomeres characterized by pathological thickening of the left ventricular wall > 15 mm unexplained by other causes such as infiltrating disease, valvular disorders, or congenital disorders [22]. Additionally, EKG findings such as left ventricular hypertrophy (LVH), inverted T-waves, left axis deviation, p-wave abnormalities are suggestive of HCM as shown in Figure 1 [22]. Common clinical manifestations of HCM include angina and syncope. Rarely, SCD and ventricular arrhythmias at rest or with exercise can occur. Among American athletes, HCM is identified in 20% of cases [23]. Specifically in NCAA athletes, 13% of deaths were attributed to HCM while 16% of deaths are from idiopathic LVH or possible HCM [11,12]. The annual incidence of SCD by HCM is 0.03–2.53 per 100,000 athletes at an annual risk of 0.02–1.27% [24]. Interestingly, in a cohort study of 1660 participants with HCM, the intensity of exercise does not correlate with a higher risk of arrhythmias or death [25]. In the study 4.6% of participants who were classified as non-vigorous achieved the primary endpoint of death, SCD, or ICD defibrillation, whereas 4.7% of those considered vigorous achieved that endpoint as well [25]. Recently, it has been proposed to loosen the restrictions placed on athletes with HCM in regards to participation in strenuous sports. The notion states that if the athlete is without any high-risk features (such as angina, prior syncope, prior SCD) and has unremarkable physiological testing then they should be allowed to participate in competitive sports [26].

When evaluating for HCM, it is important to distinguish it from an “athlete’s heart” which is a benign physiologic compensatory mechanism. In athletes, there is enlargement of the LV accompanied by increased thickness of the wall often to 13–15 mm in diameter and often homogenous instead of sporadic as seen in HCM [27,28]. In some cases, LA remodeling is also seen particularly in athletes who row or cycle. ECGs for these athletes will be abnormal in about 40% of cases, endurance athletes are the most affected [27]. On echocardiogram, features such as diastolic dysfunction and left ventricle (LV) thickness > 15 mm are highly suggestive of HCM [28]. If the diagnosis is still uncertain, then a magnetic resonance imaging (MRI) study of the heart would be the best next imaging for the diagnosis [28].

Congenital coronary artery anomalies (CCAA) are characterized by an abnormal origin or deviant course of any of the 3 epicardial coronary arteries. This category of disease can be simplified as anomalies of origin, anomalies of course, and anomalies of termination [28]. The one associated the most with SCD and myocardial ischemia is the anomalous pulmonary origin of any coronary artery [29,30]. Interarterial course of the coronary artery is also another situation that elevates risk of myocardial ischemia and SCD. A coronary artery passing through the aorta and the pulmonary artery creates a slit-like passageway which increases the risk of compression of the passing coronary artery [31,32]. Indeed, the main mechanism of SCD in those with CCAA is myocardial ischemia leading to scar tissue formation, ultimately, serving as a nidus for ventricular arrhythmias [29,32]. Among athletes, the common CCAA uncovered was anomalous left coronary artery from right sinus of Valsalva with interarterial course and anomalous right coronary artery from left sinus of Valsalva with interarterial course. Both of which made up 37% of cases [33]. In the registry, these patients died due to exercise, emotional stress, while resting and while sleeping [33]. Once an anomaly is identified or suspected, functional assessment is essential for risk stratification: maximal exercise or cardiopulmonary testing may uncover exertional ischemia or ventricular arrhythmias, while stress imaging with echocardiography or MRI can demonstrate inducible perfusion defects when ECG-based testing is inconclusive [34]. Current expert consensus emphasizes integrating detailed anatomic assessment from coronary CT or cardiac MRI (CMR) with functional data to guide decisions regarding surgical intervention and eligibility for competitive sports, recognizing that a negative stress test has limited negative predictive value in the highest-risk variants, for which sports restriction is often recommended even in asymptomatic individuals [34,35]. Some anomalies can be managed conservatively such as anomalous aortic origin of the right coronary artery; however, others are managed surgically with good outcomes [35].

ACM is an inherited cardiomyopathy characterized by fibrofatty replacement of myocardium. It is caused by mutations of the genes that encode desmosomes and other proteins in the intercellular junctions. This leads to fibrofatty replacement of normal myocardium which can serve as a nidus for ventricular arrhythmias [36]. Interestingly, exercise was found to significantly increase the risk of arrhythmias and SCD [36,37]. Additionally, even carriers of the gene are at high risk of ventricular arrhythmias with frequent exercising [38]. This carries significant weight, as having established ACM or being a carrier for ACM, even without phenotypic manifestations, would disqualify the athlete from any competitive sport [39,40]. ACM is diagnosed with the 2020 Padua Criteria; however, a new diagnostic criterion was proposed in 2024 by the European Task Force. The proposed guidelines expanded the spectrum of phenotypic presentations of ACM, as well as emphasizing the role of advanced imaging in its diagnosis [40].

Channelopathies are rare but a potential cause of SCD. It is difficult to diagnose clinically, often relying on ECGs with characteristic features to diagnose [41]. Additionally, autopsies of these hearts will appear structurally normal. Papadakis and colleagues found that in the absence of structural abnormalities, investigation of family members revealed the presence of channelopathies helping elucidate the cause of death [41].

Brugada Syndrome is an inherited autosomal dominant mutation of SCN5A which codes a subunit of the sodium channels critical for myocyte action potentials [42]. The common triggers for Brugada Pattern include fevers and rests. Often this channelopathy will present with syncope or even SCD due to VT or VF. Strenuous exercise is also shown to be a trigger for Brugada ECG changes as well [43,44]. However, current guidelines state that symptomatic individuals can resume participation in competitive sports 3 months after an ICD placement and no arrhythmias in that interim period [45]. Additionally, asymptomatic individuals, carriers can participate in most competitive sports barring those that increase core body temperature such as endurance events [45].

**Figure 2:**
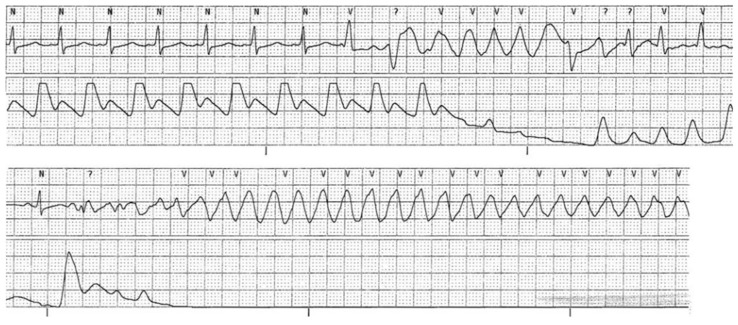
Cardiac monitoring in a patient with LV noncompaction with Long QT Syndrome complicated with Torsade de Pointes caused by a KCNQ1 mutation. N: normal sinus beat; V: ventricular tachycardia; ?; undetermined rhythm. Used with permission from Nakashima et al. [49].

Long QT Syndrome (LQTS) is defined as a QTc interval of >470 ms in men and >480 ms in women, additionally, a QTc > 500 ms is very suggestive of LQTS [46,47]. In the setting of prolonged QTc intervals, SCD can occur through polymorphic ventricular tachycardia such as Torsades de Pointes (TdP) [48]. Among athletes, the prevalence of this condition is 0.4% [48]. The main modality to diagnose this condition is with ECG. ESC guidelines recommend that all athletes with LQTS be restricted to light to moderate intensity sports [47]. Symptomatic individuals such as those with syncope or prior cardiac arrest should be barred from competitive play [47]. An ECG depicting LQTS and TdP is shown in Figure 2.

Short QT Syndrome (SQTS) does not have a formal diagnostic criteria but often these individuals have a QTc duration of <380 ms, ranging from 248–380 ms [50,51]. The prevalence of this disease is exceedingly rare, most of the patients were male and presented with SCD, syncope or atrial fibrillation [50]. The common triggers for SCD for these patients are sleep, exercise and period of intense emotions [52,53]. These athletes with SQTS are often restricted from competitive play but can participate in leisure activities [45,53].

Commotio Cordis: Traumatic causes of SCD have been identified, such as commotio cordis, which was publicly highlighted after the collapse of National Football League (NFL) player Damar Hamlin of the Buffalo Bills. Commotio cordis is caused by blunt force trauma during a 15 ms window of ventricular repolarization (i.e., upstroke of the T-wave) [54]. With precise impact timing, a premature ventricular depolarization can occur, leading to ventricular arrhythmia [54].

Left Ventricular Scars: While LV scars can occur due to myocardial ischemia secondary to CCAA, non-ischemic LV scarring due to HCM, ACM, myocarditis or sarcoidosis represents another avenue for SCD by being the origin for ventricular arrhythmias [55]. These patients will have ECG abnormalities such as low voltage, T-wave inversions and right bundle branch block [55]. SCD occurs in 5% of these athletes [56]. Strenuous exercise is often the scenario that predisposes these individuals to ventricular arrhythmias and SCD [55]. It is also important to note that scarring can also affect the right ventricle as well in cases of ARVC [57]. Interestingly, in the young cohort infiltrative or inflammatory diseases such as sarcoidosis were not found on multiple autopsies [57]. Non-ischemic LV scar is most sensitively detected by contrast-enhanced CMR as late gadolinium enhancement (LGE) [55,56,57]. It is easily missed on echocardiography due to seemingly normal wall motion [55]. As such those with a stria-like pattern of isolated LV LGE are at higher risk of complex ventricular arrhythmias and a measurable incidence of malignant events (sustained VT, appropriate ICD therapies, or sudden death), whereas small junctional or “spotty” LGE at the right ventricle (RV) insertion points appears more often as a benign adaptation with lower arrhythmic risk [55]. Diseases such as this underscore the importance of multimodal imaging when evaluating cardiac diseases in young athletes.

Drug induced: Toxicology positive SCD represents another facet of this phenomenon. While toxicology positive SCD has lower prevalence in athletes compared to nonathletes. The common drugs found in athletes were cannabinoids, psychotropic, and antiepileptic agents. Cannabinoids can cause tachycardia and induce a type 1 Brugada pattern [58]. Additionally, polypharmacy with psychotropic medications can lead to QT prolongation, predisposing the user to polymorphic ventricular tachycardia [59].

**Figure 3:**
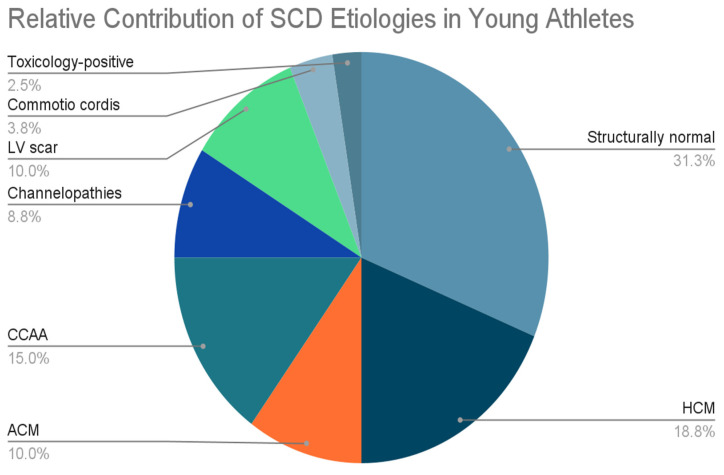
Pie chart summarizing the prevalence of different etiologies of sudden cardiac death. HCM: hypertrophic cardiomyopathy, CCAA: congenital coronary artery anomalies, ACM: arrhythmogenic cardiomyopathy, LV: left ventricle.

Overall, structurally normal hearts (idiopathic or primary arrhythmic mechanisms) now account for the largest proportion of SCD cases [11,12,20,21]. HCM remains the most common structural cause, especially in the U.S [17,18,23], while ACM/ARVC is more prevalent in European cohorts [19,20,37,38]. CCAA are a significant contributor, particularly anomalies with interarterial courses, which can precipitate ischemia and SCD [29,30,31,32,33,34,35,36]. Inherited channelopathies—including Brugada syndrome, LQTS, and SQTS—are less frequent but clinically important, often presenting in structurally normal hearts [42,43,44,45,46,47,48,49,50,51,52,53]. Other rarer etiologies include traumatic causes such as commotio cordis [54], non-ischemic or ischemic LV scarring [55,56,57], and toxicology-positive events [58,59]. A summary of the prevalence of the different etiologies is summarized in Figure 3. This figure emphasizes the heterogeneity of SCD in young athletes and underscores the need for tailored screening, multimodal imaging, and individualized risk stratification.

### 2.3. PPCS for Competitive Sports

The concept of PPCS for young athletes (<35 years) emerged in the 1980s during the 16th Bethesda Conference, sponsored by the American College of Cardiology (ACC), which proposed guidelines on disqualification criteria for athletes in competitive sports [60]. These initial guidelines were not widely implemented, but the issue gained national attention following the tragic death of Hank Gathers in 1990 [61]. This event highlighted the limitations of relying solely on history and physical examination for detecting underlying cardiovascular abnormalities.

### 2.4. From Guidelines to Policy

In 1996, the American Heart Association (AHA) with the ACC released guidelines emphasizing history and physical examination without electrocardiogram (ECG), a stance reaffirmed in 2007 in an exert consensus statement [62,63]. Years after, the question of the role of ECG was once again revisited by the AHA/ACC, the authors affirmed their predecessor’s statement in advising against universal ECG screening in young athletes (ages 12–25 years) in competitive sports. In their statement, the authors argued that there is inadequate evidence supporting the benefit of routine use of ECG as firstly there is no national database or federally enforced mandatory reporting system implemented in the United States [64]. The lack of a centralized database, leaves the true incidence of SCD unclear [64]. Moreover, the authors noted that a sufficiently powered randomized clinical trial investigating mortality in populations with routine ECG screen compared to those without is “impractical” [64]. The lack of data on mortality benefit combined with logistical and economic problems, ultimately, making it unfeasible when applied to the general population [64].

Despite these recommendations, states and local communities have passed or advocated for laws mandating ECGs in PPCS screening. On 25 June 2025, Florida enacted the Second Chance Act, requiring all students (Pre-K to 12) to obtain at least one ECG prior to sports participation and prohibiting play for students with abnormal results until cleared medically [65,66]. This legislation, inspired by the death of Chance Gainer, reflects a societal and legal emphasis on risk mitigation, even in the absence of conclusive trial data, illustrating the interplay between public health policy and clinical evidence [65]. However, Florida is not the only state to pass such a bill as, in 2019, Texas passed Cody’s Law [67]. Uniquely Pennsylvania’s Peyton’s Law does not utilize the power of the state to compel ECG screenings [68]. Instead, the law requires the Pennsylvania Interscholastic Athletic Association (PIAA) documents require a section explaining the importance of ECGs in detecting abnormal heart disease that could predispose the student to SCD and allow for an option for the family of the student to request an ECG from a physician at their own expense [68]. The law also requires any student with signs or symptoms of heart disease or SCD to be prohibited from play or practice. Medical clearance from a physician, nurse practitioner or cardiologist is required for the barred student to play [68].

The topic of PPCS raises several questions regarding patient autonomy. As stated previously, routine PPCS has the risks of false negatives and false positives. False disqualifications, defined as the improper disqualification of a healthy athlete, is a possibility which can create anxiety and negatively impact the athlete’s livelihood. However, the incidence of these false disqualifications is unclear and has not been extensively studied. One study estimates that 0.2% of athletes were disqualified for “potentially” fatal cardiac disease [69]. While Texas passed a mandatory ECG in 2019, there has been no reported suits or claims of false disqualification. From a medico-legal perspective, the focus has been on the “duty of care” of the physician and the school as opposed to the patient’s autonomy [70,71]. Negligence in this scenario is both tragic and costly, as for example, following Gathers’ death his family sued his cardiologists and university for millions which was later settled for $545,000 [72]. For physicians, the duty of care is an extension of the classic physician ethics of beneficence and non-maleficence. Because of duty of care, schools can ban student athletes from playing should there be a concern of lethal cardiac disease. One of the earliest case was Larkin v. Archdiocese of Cincinnati (1990) ruled in the U.S District Court for the Southern District of Ohio [70,71]. In this case, the court ruled that students with diagnosed heart disease do not have the “compelling right” to participate in school sponsored sports without proper medical clearance [70,71]. Years later, Nick Knapp suffered from cardiac arrest while playing basketball. Despite having a defibrillator and clearance from his physicians, Northwestern University barred him from play. Despite winning his earlier lawsuit against the school, the decision to unbar him was overturned by the 7th Circuit Court of Appeals in Knapp v. Northwestern University (1996) [73]. In this ruling, the court determined that schools can rely on their own medical experts to determine if a student is safe to participate in school-sponsored sports [71,73]. Interestingly, both Knapp and Larkin invoked the Rehabilitation Act of 1973 (a law which prevents discrimination on the basis of disability) as the spearhead of their argument [70,71,73]. Both times, the courts ruled that the school’s decision to ban these athletes did not violate the Rehabilitation Act. Overall, the safety of student athletes is the responsibility of both the schools and the physicians. Current legal precedent gives the school the final decision on whether a student athlete can participate in sports.

In Europe, the first PPCS guidelines were issued by the European Society of Cardiology (ESC) in 2005 [74]. Unlike the AHA/ACC, the ESC strongly supports routine ECGs as part of screening, based on observational evidence, primarily the landmark Italian study by Corrado et al., which evaluated mortality trends before and after nationwide ECG screening (1979–2004) [75]. The study demonstrated a reduction in SCD incidence from 3.6–4 per 100,000 person-years pre-screening to 0.43 per 100,000 post-screening, with most deaths occurring in males aged ~23 years [76]. The authors attributed this decline to early identification of structural and electrical cardiac abnormalities, illustrating a methodologically plausible link between ECG screening and mortality reduction in high-risk athletic populations [75]. Based on these findings, the ESC recommends ECG inclusion to detect hypertrophic cardiomyopathy, ACM, and channelopathies such as long QT syndrome [74,76].

Nowadays, Italy and Israel are the only countries with nationally enforced universal cardiac screening for all young athletes [77]. Several European countries such as Germany, Sweden, Norway and France created universal cardiac screening policies only for professional athletes such as soccer players [78]. Conversely, Denmark has rejected any form of universal PPCS screening [77,78]. The Danish Society of Cardiology (DSC) rejects universal PPCS screening for young Danish athletes, going against the ESC and AHA/ACC recommendations. Gaarsdal Holst et al. studied the incidence of SCD amongst all young athletes during 2000–2006 [79]. In the study population, the incidence was 1.21 per 100,000 person years. Compared to the general population which had an incidence of 3.76 per 100,000 person years [79]. Within the SCD group, the most common causes were ACM, structurally normal heart and coronary artery disease [79]. In the end, the authors questioned the benefit, if any, of universal PPCS screening for the Danish population [79]. This suggests that selective screening, informed by epidemiologic risk profiles, may offer a cost-effective alternative in populations with lower baseline incidence.

### 2.5. Understanding Guideline Divergence and Disqualification Criteria

The divergence between U.S. and European recommendations reflects methodological, epidemiological, and sociocultural factors, as well as fundamentally different philosophies regarding athlete disqualification. In the U.S., the historically low absolute risk of SCD in young athletes, the absence of a centralized reporting system, and the impracticality of large, randomized trials have contributed to a more conservative approach to both ECG screening and athlete disqualification [61,62,63]. U.S. guidelines aim to minimize unnecessary removal from sport, emphasizing shared decision-making and individualized assessment to avoid the psychosocial and financial consequences of disqualification in low-risk scenarios as demonstrated through US state initiatives to mandate ECGs [63,65,67,68]. Consistent with this philosophy, U.S. recommendations generally reserve permanent disqualification for athletes with uncontrolled ventricular arrhythmias, high-risk structural disease, syncope of cardiac origin, or persistent markers of electrical instability, while allowing conditional participation with specialist oversight when risk is uncertain [63,65,67,68].

**Figure 4:**
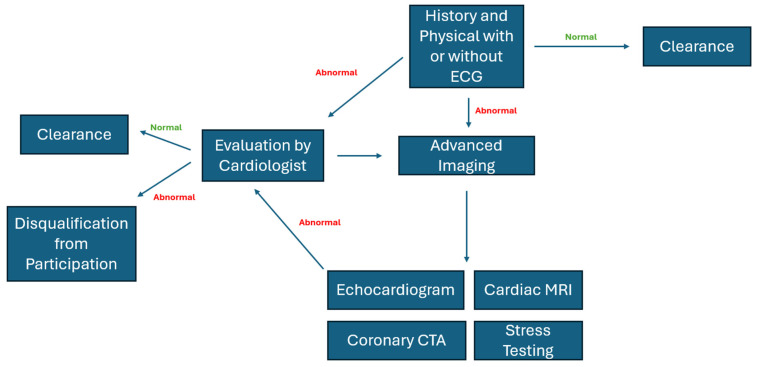
Algorithm for PPCS. CTA: computerized angiography.

In contrast, European recommendations operate within centralized health systems supported by long-term registry data, greater historical precedent for mandatory ECG screening, and an ethical framework that prioritizes maximal SCD prevention—even accepting higher false-positive and disqualification rates as an acceptable trade-off [74,75,76,77]. As a result, European criteria more readily endorse temporary or permanent disqualification for athletes with HCM with any major risk marker, ACM regardless of symptomatic status, CCAA with interarterial or intramural courses, LQTS or Brugada syndrome with abnormal ECG patterns, and any evidence of exercise-induced arrhythmias [74,75]. Sociocultural expectations also differ: European policy generally favors precautionary exclusion when diagnostic uncertainty exists, whereas U.S. policy emphasizes athlete autonomy and risk tolerance, often permitting continued participation under expert supervision unless risk is clearly elevated [74,75]. A summary of the qualification and disqualification algorithm is shown in Figure 4.

In summary, although PPCS cardiovascular evaluation is globally recognized as essential, the thresholds for disqualification vary according to regional epidemiology, healthcare infrastructure, and differing views on acceptable risk. Moving forward, refinement of risk stratification tools, integration of national outcome registries, and selective ECG-based screening may help align disqualification decisions with individualized risk while preserving athlete participation whenever safely possible. A summary of the key differences between the AHA/ACC and ESC guidelines are summarized in Table 1.

## 3. Discussion: Comparison of AHA/ACC 2014 and ESC 2020 PPCS Screening Guidelines

Both large organizations agree that history and physical (H&P) is a critical part of the PPCS screening. In 2014, the AHA/ACC expanded screening criteria to a 14-element tool, incorporating personal and family history alongside physical exam components [64,80]. The widely used PPCS Physical Evaluation monograph, endorsed by multiple medical and sports organizations, covers a comprehensive PPCS physical beyond cardiovascular issues but aligns with AHA cardiac screening guidelines. A major challenge in the U.S. is the compliance gap with AHA/ACC screening recommendations [81]. Ideally, screening should occur at least six weeks before participation, with re-evaluation every year, and primary care physicians should conduct these evaluations, involving parents to address discrepancies in reported history [82]. Any abnormalities on the initial assessment is required to further evaluate with advanced imaging and proper medical clearance is required before clearance to participate in competitive sports [62,64,83].

A criticism of an H&P only approach to PPCS screening is the high false negative rates and poor positive predictive value [84]. When screening high school athletes (mean age 16 year) with the AHA-14 questions, sensitivity was found to be 18.8% with a specificity of 75.1% [84]. On the other hand, H&P with ECG, sensitivity and specificity sharply rose to 87.5% and 97.5% respectively [84]. Furthermore, H&P with ECG had a positive predictive value of 13.3% and a negative predictive value of 99.9% [84]. Indeed, another study found that the addition of ECG can increase the probability of detecting a cardiac disease by up to 6 times [85]. Furthermore, critics also argued that H&P alone can lead to unnecessary referrals and miss up to 66% of cardiac disease that could have otherwise been detected with ECG [86]. These questions rely on complete honesty and transparency for the athlete in question. A difficult situation considering that a potential career and livelihood are at stake [77,87]. However, athletes report no increase in stress/anxiety when undergoing the PPCS process. As expected, anxiety and stress was higher in those who had a true positive for an underlying cardiac issue [87].

Because of the pitfalls of performing an H&P alone, advocates argue that ECGs are a critical and necessary component of the PPCS screening. Central to their argument is the superior diagnostic accuracy of ECGs in detecting common culprits of SCD such as ACM, HCM and even rare channelopathies (of which are unable to be detected by H&P alone) [83,88,89,90,91,92,93]. One of the earliest endorsements of ECGs comes from Corrado et al. In his study, the routine use of ECGs combined with other workup successfully diagnosed 22 athletes with HCM [93]. Following the disqualification of these individuals, none of them died at the end of the observation period [93]. The authors concluded that by disqualifying athletes with the confirmed diagnosis of HCM, in turn, will reduce mortality [93]. Additionally, a meta-analysis found that ECG alone is five times more sensitive than history, ten times more sensitive than physical exams and carries a superior positive and negative likelihood ratio compared to H&P alone [88].

In response, experts in the AHA/ACC argued that there is no mortality benefit for ECGs [64]. They pointed out that the landmark 2005 Corrado et al. study which demonstrated a mortality benefit of combined H&P and ECG PPCS screening is not generalizable as the study only looked at the Veneto region (which constituted 9% of the overall Italian population) also the results of the study could not be replicated around the world [63,64]. Maron and colleagues compared SCD incidence in athletes in Minnesota and Veneto from the 1980s to the early 2000s [94]. Both of the examined populations have similar population count and are ethnically homogenous [94]. Over the study period, 55 SCD events occurred in Italy while 22 deaths occurred in Minnesota. The author attributed the elevated mortality on the Italian side to the early phases of screening from 1982–1985 [94]. However, following that period both populations had comparable mortality rates. Despite enforcing a universal PPCS in 1997, Israel has also not seen a significant mortality benefit as 11 SCD events were reported prior to the law’s passage, and 13 deaths were reported after it [95]. It is worth noting that this study was heavily criticized for its methodological flaws and reliance on media reporting [96,97]. An H&P alone is only good at identifying diseases that present with heart murmurs or symptoms relating to connective tissue disease [77]. To date, there have been no studies in the United States that found a mortality benefit with the routine use of ECGs. Now that Florida has recently passed a law requiring the use of ECGs for PPCS assessments, there is an opportunity to investigate potential mortality benefits for the Floridian population. However, as mentioned above, RCTs into this topic would be beneficial from a data collection standpoint but unviable in terms of logistics and ethics [95]. Nevertheless, in 2014 the AHA/ACC guidelines softened their stance on the role of ECGs, stating that it can be considered in select cases as a first line diagnostic test [64].

However, ECG cannot detect all conditions, such as anomalous coronary artery origin and coronary artery disease (CAD), highlighting the need for complementary imaging modalities. Interobserver variability remains one of the most significant weaknesses of an ECG guided PPCS protocol [98,99]. This phenomenon is observed in both sports physicians and cardiologists alike [98,99,100]. High false positive rates between 5–46% have been noted [77]. The creation of international guidelines such as the 2013 Seattle Criteria and the Refined ESC Criteria helped lower the variability [95]. When using guidelines the false positive rate will decrease to 1.3–6.8% [77]. Variability amongst false positive rates depends on the prevalence of cardiac pathology in the screened population as well as the level of experience of the interpreting physician. Nevertheless, the consequences of a misinterpretation of an ECG can lead to costly cardiac workup or even death on the other hand, false positives in ECG interpretation can be costly and potentially disqualify a healthy athlete [77].

The ethical considerations and the importance of false positives surrounding cardiovascular screening in young athletes for the sole purpose of preventing SCA and SCD creates a complex interplay and balancing act between the goal of avoiding premature death in this population while still respecting athlete autonomy and mitigating the potential harms of false positives which can cause unwanted stress, further invasive and costly studies, and loss of play time in the interim [91]. At times, there is insufficient evidence and inconsistency in organizational guidelines pertaining to cardiovascular screening protocols in young athletes which can lead to drastic inequalities and outcomes which can negatively impact resource allocation and the value-based thresholds for what is deemed as pathological versus benign [91].

Current trends focus on improving accuracy through artificial intelligence (AI)-driven ECG analysis and genetic testing, which may help refine screening methods while minimizing unnecessary testing and misdiagnoses, potentially bridging the gap between European and American approaches in the future [101,102]. However, its use is limited to the acute setting in specific and controlled circumstances and has the risk of bias and inaccuracies [101,102].

### 3.1. Serial ECGs

Repeat assessments of athletes is recommended by both the AHA/ACC and ESC guidelines. The AHA recommends annual or biannual examination of all young athletes, similarly the ESC recommends annual examinations for athletes especially those with diagnosed heart disease prior to play [64,83]. While there is limited data on whether serial ECGs have a mortality benefit (i.e., detecting a missed diagnosis, monitoring for progression), nevertheless, guidelines support annual PPCS screening with history and physical with or without an ECG. Indeed, Pelliccia et al. found that athletes who present with abnormal ECG abnormalities such as T-wave inversions but no apparent cardiac disease will end up developing clinically significant symptoms such as syncope or death [103]. Eventually, the diagnosis of cardiomyopathy is made after serial ECG and echocardiograms [103]. The authors noted that non-specific abnormalities on ECGs can be the first indication of a genetic heart disease [103]. However, there remains a lack of data on the long-term mortality benefit of the continued use of annual ECGs in PPCS. Furthermore, there is also a lack of data on whether annual ECGs (on asymptomatic low risk athletes) is superior to other PPCS strategies such as a one time ECG on initial screening then PPCS without ECGs for asymptomatic low risk athletes.

### 3.2. Role of Advanced Imaging in Screening

Beyond history, physical examination, and ECG, advanced imaging techniques such echocardiography, CMR, and CT have gained prominence for follow-up evaluations when initial screenings are abnormal or inconclusive [104]. These imaging modalities are essential for differentiating between physiological adaptations—such as the “athlete’s heart”—and pathological conditions like hypertrophic cardiomyopathy or myocarditis [104]. Echocardiography plays a crucial role in confirming diagnoses, particularly for structural heart diseases like HCM, but is not used routinely for screening due to cost and accessibility constraints. However, emerging echocardiographic techniques have shown potential in detecting myocarditis, right ventricular remodeling, and coronary anomalies, which are significant contributors to SCD in athletes [105]. In select cases, CMR provides superior tissue characterization and is particularly useful in diagnosing ACM but remains cost-prohibitive and impractical for widespread PPCS screening. Similarly, coronary CT angiography (CCTA) is emerging as a valuable tool for evaluating master athletes (>35 years) at high risk for CAD, though routine stress testing is not recommended due to high false-positive rates [106].

### 3.3. Echocardiography

Echocardiography is one of the most ubiquitous cardiac imaging modalities available. While inexpensive, assessable, and physiologically harmless, the main drawback of echocardiograms is that it is operator dependent in both the gathering and interpreting of the images [107,108,109]. When combined with the standard H&P and ECG protocol, the addition of echocardiogram adds about 10% additional detection of the rate of underlying cardiac diseases otherwise missed on the standard PPCS screening protocol [108]. Because of its excellent utility, it is often used as the first line advanced imaging modality for any suspected cardiac pathology [83,110]. These range from evaluating murmurs, abnormal ECG findings (T-wave inversions, axis deviations, or any other arrhythmias), family history of SCD, as well as symptoms suggestive of cardiac etiology [107,108,109,110]. However, echocardiograms may be inconclusive or equivocal depending on operator skill or underlying pathology [107]. As will be discussed later, it should be combined with other advanced imaging to better delineate the underlying disease, if any. Guidelines from both sides do not recommend echocardiograms as part of the routine PPCS battery [64,83]. Although this assertion is being challenged as there are athletes who are asymptomatic but have underlying diseases such as bicuspid aortic valve, hypertrophic cardiomyopathy, and mitral valve prolapse [111].

### 3.4. Exercise Stress Testing

Exercise stress testing and stress testing with imaging are two imaging methods useful at predicting mortality among asymptomatic individuals. Often the greatest risk factor for cardiac mortality is exercise intolerance [107,112]. In young athletes, this risk factor is not applicable [107]. There is a scarcity of data regarding the routine use of exercise stress testing in this population, Sofi et al. is one of the few who did so. Looking at the Italian athlete population, each participant underwent a resting and exercise ECG prior to competitive play [113]. At the end of the follow up, of the athletes who were disqualified from play 79.2% had normal resting ECGs [113]. The disqualifying features on the exercise ECGs include ST-T segment changes suggestive of coronary artery disease and ventricular tachycardia [113]. The mean age of the disqualified athletes was 30.7 years [113]. Therefore, in the young (i.e., high school or college age) athlete population especially those without symptoms the use of exercise ECGs is not recommended as part of the standard PPCS screening [107,114].

### 3.5. Coronary Computed Tomography

For young athletes (<35 years old), coronary artery testing may seem low yield as coronary artery disease (CAD) is one etiology of SCD in older/master athletes (>35 years). However, as described earlier, CCAA is an important cause of SCD in young athletes and CCTA is the preferred imaging modality for diagnosing this congenital anomaly. The typical symptoms which may prompt coronary artery evaluation would be angina, syncope, dyspnea on exertion, and palpitations [115]. While echocardiogram is used as the first line imaging modality for suspected anomalous coronary arteries, as it can visualize the ostia and first tracts of the coronary arteries [116]. The drawbacks are that its sensitivity is highly variable mainly due to operator skill [116,117]. Additionally, myocardial bridging is difficult to visualize on echocardiography [118]. CCTA allows for complete evaluation of the anatomy of the anomalous coronary artery. When compared to invasive angiography, multislice CCTA was superior in detecting all coronary artery anomalies whereas invasive angiography was able to correctly detect 53% of coronary artery anomalies [119,120]. Furthermore, CCTA can evaluate for systolic compression of the coronaries, slit like openings, and intramural course of the arteries [121]. The sensitivity of CCTA varies based on the slice acquisition, early investigations into CCTA were conducted with 4-slices which first demonstrated the feasibility of the imaging modality [122]. With 16-slice CCTA, the sensitivity 83–98% with specificity of 96–98% [122]. Moreover, with 64-slice CCTA, the sensitivity ranges from 73–100% and specificity 91–97% [122]. Identifying all the potential mechanisms of myocardial ischemia facilitates surgical management of CCAA [117]. There are drawbacks to CCTA, one is radiation exposure [122]. To reduce radiation dosages, ECG gating protocols were introduced [120,122]. In this method, radiation is only given at specific parts of the cardiac cycle instead of being administered continuously [123]. It was found that radiation dosages ranged from 1.1–3.0 mSv to gather quality images [123]. Whereas, without ECG-gating, the average radiation dose was 21.4 mSv [123]. However, for this method to be viable, heart rates must be <63 BPM and no atrial fibrillation must be present [123]. In this study, 75% of patients received beta-blockers [123]. Overall, CCTA is the gold-standard for diagnosing CCAAs and this study should be pursued in young.

### 3.6. Cardiac MRI

CMR is a non-invasive imaging study that does not utilize radiation. One of the advantages of CMR is its ability to characterize myocardium, making it effective in evaluating for cardiomyopathies and LV scars [124,125]. Additionally, it also has the ability to assess cardiac function in cases where echocardiography is equivocal [125]. MRI protocols such as T1 and T2 signal mapping are both utilized to characterize inflammatory or edematous changes in the myocardium [125]. LGE is another diagnostic study performed on MRI [125]. LGE is excellent at uncovering scar tissue as it contrasts washout of gadolinium contrast between healthy myocardium and scar tissue [125]. The latter has slower clearance [125]. CMR is not used as a first line imaging study for PPCS however, similar to the previously mentioned imaging modalities CMR is often used as a follow up study to diagnose cardiomyopathies. 

## 4. Discussion: The Complete Evaluation of Cardiac Structure, Function and Viability

With many unique imaging modalities available, the pathway on what to use and when to use depends on clinical suspicion and presentation. Current guidelines recommend against the use of routine non-invasive imaging for PPCS [126]. As such the decision to follow up and evaluate suspected cardiac pathology is based on the H&P with or without ECGs [126]. As described earlier, for any abnormality on H&P such as exertional chest pain, dyspnea on exertion, or unexplained syncope, a transthoracic echocardiogram should be the first line imaging study thanks to its accessibility but also its ability to evaluate myocardial function and structure, valvular function and integrity, and the origins of coronary arteries [126]. Certain cardiac pathologies such as valvular disorders and hypertrophic cardiomyopathy can be readily diagnosed with echocardiogram alone without the need for additional imaging. However, there will be cases where echocardiograms are inconclusive or equivocal [126]. In these scenarios, combining different imaging would be required to elucidate the underlying pathology and evaluate myocardial viability [127,128,129]. A summary of the advantages and disadvantages of various advanced imaging modalities and tests is depicted in Table 2.

For example, in the case of syncope, the differentials can range from channelopathies, outflow obstruction, ischemic cardiomyopathy, infiltrative cardiomyopathy, or myocardial scarring. If ECGs and echocardiograms are inconclusive, then follow up imaging with CMR and CCTA would be ideal to better understand the underlying pathology [128]. Firstly, CCTA is the test of choice for ruling out CCAA. CMR is excellent at evaluating for not only assessing myocardial function but also in the evaluation of myocardial scarring, ischemic cardiomyopathy and infiltrative cardiomyopathies such as ACM [128]. In CMR the use of LGE and end-diastolic wall thickness are both useful markers for myocardial viability [127,128]. The presence of the former and a cutoff valve of <5.5–6.0 mm for the latter are signs of non-viable myocardium [128]. If there is ischemic cardiomyopathy or LV scars, then combining these two tests can not only diagnose the underlying pathology but also uncover the mechanism which will aid in formulating management strategy and influence the decision making for return to play. Lastly, the addition of exercise stress testing with or without imaging is vital test for elucidating cardiac pathologies that occur during physical activity. This test alone has poor sensitivity and specificity at 66% and 61% respectively [129]. When combined with imaging the diagnostic accuracy improves with a sensitivity and specificity of 81% and 85% respectively [129]. Because of its limitations, exercise stress testing is best combined with other non-invasive imaging studies to achieve the best diagnostic results [126,129]. A summary of the different evaluations for various cardiac pathologies is summarized in Table 3.

### 4.1. Economic Impact

Cost-effectiveness remains a central debate in PPCS cardiovascular screening, particularly regarding ECG-based approaches. One cost-analysis estimates that ECG screening will cost $76,000 per life saved with an cost of $199 per athlete [130]. H&P alone has a cost per life saved of $84,000 [130]. Another study concluded that routine ECGs program over 20 years would cost between $51 and $69 billion with estimated cost per life between $10.6 million to $14.4 million over that theoretical timeframe [131]. At the University of Virginia, a 5 year mandatory ECG program cost $894,870 with the cost of H&P alone being $68,745 or $343,725 per finding [132]. With ECG added to H&P, the cost per finding was similar at $68,893 or $551,145 [132]. In Italy, a nationalized PPCS with ECG was found to cost €4 million with a cost of €69.80 per athlete and €156.204 for the diagnosis of a cardiovascular disease which can lead to SCD [133]. The methodologies of these studies contribute to the wide differences in cost of the program and cost per athlete. These studies examined different populations (Virginians vs. Italians), in different healthcare systems and in different time periods. There is a lack of studies on a nationalized ECG mandated PPCS in the US because there is no law requiring this protocol unlike in Italy. Additionally, the existence of the cost burden and its relative financial spectrum on cardiovascular screening is related to the healthcare system variability that is noted in many parts of the world (i.e., universal healthcare versus private insurance companies). Government funded healthcare programs approach to SCD in athletes focus on two primary, debated strategies: prevention via universal EKG screening and emergency, on-site response protocols such as CPR and AED training [134]. Nevertheless, these studies agree that the addition of ECG to PPCS will dramatically lower the cost per life saved. However, critics argue that the low incidence of sudden cardiac death in young athletes limits the overall impact of screening on mortality reduction [77]. Furthermore, healthcare insurance in the US is not arranged to cover the use of ECGs in routine PPCS. Indeed, ECGs were ordered less for those with lower socioeconomic status and in rural settings [135]. Additionally, the uncertain timing of disease onset further complicates screening effectiveness, as a single evaluation may not capture evolving cardiac pathology, leading to inconsistent screening practices. Both the US and, to some extent Europe, are diverse places with unique populations distributed in different geographical areas (urban, suburban, rural). Because of their constituents’ unique demographics and health needs, implementing a universal mandatory ECG law would be very difficult from a policy and economic standpoint. As demonstrated, laws requiring ECGs are best implemented by individual US states and European countries. Given these financial and logistical concerns, a balanced approach incorporating targeted ECG screening and selective use of advanced imaging may offer the most practical and cost-effective solution for identifying high-risk individuals while minimizing unnecessary expenditures.

### 4.2. Limitations

This review, even with additional manual searches to augment data organization and overall conclusions, is still subject to selection bias, data heterogeneity, and author, potentially lending to some overgeneralizations. Additionally, there remains a lack of data regarding mortality benefit, sensitivity and specificity of ECGs in different populations (such as elementary or college aged athletes) and alternative PPCS strategies. Therefore, more research needs to be done to explore these topics to better guide recommendations and policy.

## 5. Conclusions

Both the AHA/ACC and ESC guidelines have different approaches to routine PPCS in young athletes. The role of ECG being the main point of contention between the two. While ECGs can provide additional diagnostic sensitivity for underlying cardiomyopathies and arrhythmias, it does have drawbacks such as false positives or false negatives due to observer variability. Nevertheless, should any suspicion of cardiac disease be held there are many secondary imaging modalities available to evaluate a disease of concern. The integration of targeted ECG screening with echocardiography and advanced imaging in select cases can enhance diagnostic accuracy while balancing cost-effectiveness and accessibility. Because of the lack of cohesive guidelines, differences in healthcare systems and population characteristics, many countries and US states are implementing different approaches to routine PPCS for not only young athletes but also older and professional athletes as well. Additionally, newer technologies such as AI driven ECG analysis has potential to reduce ECG interpretation variability. To improve outcomes and facility policies, the routine use of ECGs should be encouraged. As the last AHA/ACC guidelines were made in 2014, this topic should be re-evaluated by these organizations in light of new evidence supporting the role of ECGs. Although more literature has been published regarding this topic, additional data on mortality would be needed to guide recommendations. Furthermore, new diagnostic tools such as genetic testing and AI-assisted ECG interpretation are being investigated but not yet implemented. Lastly, policymakers should be involved in not only raising awareness of this issue but also drafting initiatives designed.

## Figures and Tables

**Table 1:** Summary of key differences between the AHA/ACC (U.S.) and ESC (European) PPCS recommendations, with the Italian model included due to its historical influence. The comparison focuses on (1) recommended PPCS components, (2) screening interval, (3) required expertise, (4) mandated versus optional ECG use, and (5) follow-up testing pathways. Terminology is standardized throughout: PPCS refers to preparticipation cardiac screening for athletes < 35, master athletes refers to individuals > 35, and students Pre-K–12 follow standard U.S. PPE protocols.

Feature	AHA/ACC (U.S.)	ESC (Europe)	Italian Protocol
Target Population	PPCS for competitive athletes < 35; “master athletes” > 35; applies to students Pre-K–12 for basic PPE	PPCS for competitive athletes < 35; exercise guidelines for master athletes > 35	Mandatory PPCS for competitive athletes < 35
Recommended Components	H&P only using 14-element AHA tool ECG optional/selective	H&P + mandatory 12-lead ECG as first-line	H&P + mandatory ECG ± exercise testing, long-standing protocol
Screening Interval	Annual or biannual; ideally ≥6 weeks before participation	Annual assessment for all athletes; shorter intervals for those with disease	Annual mandatory assessment
Required Expertise	Primary care physicians; cardiology referral for abnormal findings	Sports cardiology expertise preferred; standardized ECG training encouraged	Certified sports medicine physicians with cardiology support
ECG Use	Optional: “may be considered” in selected cases	Mandated for all competitive athletes < 35	Mandated since 1982 (national law)
Follow-up Testing	Selective use of echocardiography, CMR, stress testing when H&P/ECG abnormal	Echocardiography, CMR, CPET, genetic testing per ESC cardiomyopathy/channelopathy guidelines	Stress testing widely used; strong emphasis on restricting at-risk athletes
Philosophical Approach	Minimize false positives and avoid unnecessary disqualification; autonomy-focused	Maximize SCD prevention even if false positives/disqualification are higher	Aggressive identification and disqualification of at-risk athletes, historically reducing regional SCD

**Table 2:** Summary of the indications and disadvantages of different imaging and testing modalities. TTE: transthoracic echocardiography.

Modality	Availability	Indications	Cons
History & Physical Examination	Ubiquitous	May be present as a routine part of preparticipation cardiac screening Symptoms suggestive of cardiac etiology (chest pain, dyspnea on exertion, unexplained syncope, palpitations) Family history of sudden cardiac death	Low sensitivity for detecting asymptomatic conditions. Low sensitivity for detecting inherited conditions. Poor predictive value compared to ECG and imaging modalities.
Electrocardiogram	Ubiquitous	Symptoms suggestive of cardiac etiology Family history of sudden cardiac death Abnormalities on ECG (LVH, axis deviations, T-wave inversions)	False positives remain a challenge, Requires trained specialists, Not reliable for detecting congenital coronary anomalies. Cost-effectiveness concerns.
Echocardiography	Ubiquitous	Symptoms suggestive of cardiac etiology Abnormal or inconclusive TTE	Operator-dependent accuracy. Not cost-effective for mass screening. Limited in detecting congenital coronary anomalies. False positives in trained athletes.
Coronary Computed Tomography Angiography	Limited	Symptoms suggestive of cardiac etiology (specifically concerning for ischemia) Abnormal or inconclusive TTE	Diagnostic utility limited to coronary artery anatomy evaluation Radiation exposure
Cardiac Magnetic Resonance Imaging	Limited	Symptoms suggestive of cardiac etiology Abnormal or inconclusive TTE	Requires specialized training Time-consuming. High cost burden. Impractical for mass screening.
Exercise Stress Testing	Ubiquitous	Symptoms suggestive of cardiac etiology Family history of sudden cardiac death	Poor sensitivity and specificity when done without imaging Time-consuming May be uncomfortable or intolerable if symptomatic
Genetic Testing	Limited	Diagnosed inherited cardiomyopathy or channelopathy	Cost-effectiveness concerns

**Table 3:** Summary of recommended workup for different etiologies of sudden cardiac death. HCM: hypertrophic cardiomyopathy, CCAA: congenital coronary artery anomalies, ACM: arrhythmogenic cardiomyopathy, LQTS: long QT syndrome, SQTS: short QT syndrome, LV: left ventricle. MRI: magnetic resonance imaging, CT: computed tomography, RBBB: right bundle branch block, ECG: electrocardiogram, LGE: late gadolinium enhancement.

Etiology	ECG Detectability	Imaging Modality	Notes
HCM	LVH, T-wave inversion, axis deviation	Echo (LV thickness, diastolic dysfunction), MRI if uncertain	Distinguish from athlete’s heart
CCAA	Exercise-induced ischemia or arrhythmia (may be subtle)	Coronary CT, stress echo or MRI	Functional + anatomic assessment needed
ACM	Ventricular arrhythmias	Cardiac MRI (fibrofatty replacement, LGE)	ECG may miss early structural changes
Brugada Syndrome	Coved ST elevations in V1–V3	Limited imaging utility	ECG is primary
LQTS/SQTS	QTc prolongation/shortening	Limited imaging utility	ECG is primary
LV Scars (non-ischemic)	Low voltage, T-wave inversions, RBBB	Cardiac MRI with LGE	Echo may miss subtle scars
Commotio Cordis	Acute ventricular arrhythmia	Not applicable	Trauma-induced
Drug-induced	QT prolongation, Brugada patterns	Not primary	ECG critical for monitoring

## Data Availability

Data from this manuscript was obtained through PUBMED and Google Scholar.
